# Analysis of medical profession and time of occupa6onal exposure among health workers in RSUD Dr. Saiful Anwarmalang

**DOI:** 10.1017/ash.2025.108

**Published:** 2025-09-03

**Authors:** Milanitalia Gadys Rosandy, Dewi Indiastari, Heri Sutanto, Didi Candradikusuma, Niniek Budiar C

**Affiliations:** * Tropical Medicine and InfecCous Disease Subspeciality Program, Faculty of MedicineUniversitas Brawijaya; ** Department of Internal Medicine, Tropical Medicine and InfecCous Disease, Faculty of MedicineUniversitas Brawijaya/Dr. Saiful Anwar Hospital, Malang, Indonesia

## Abstract

**Background:** Occupa(onal blood and body fluid exposure is one of the major public health problems in healthcare workers (HCW). This condi(on had the risk of transmission of infec(ous diseases. Educa(on level is oEen considered a key factor influencing the frequency and dura(on of exposure. This study inves(gates the correla(on between medical profession and the (me of occupa(onal exposure among health workers at RSUD dr. Saiful Anwar Malang from 2020 to 2024. **Objective :** The primary objec(ve of this study is to determine whether there is a significant correla(on between the educa(on level of health workers and their exposure, with the aim of iden(fying poten(al areas for interven(on to reduce occupa(onal hazards. **Methods** : A cross sec(onal study from health workers from at RSUD dr. Saiful Anwar Malang. Data included gender, medical profession (nurse, resident, doctor, medical student, cleaning service), and (me of exposure (work hour, duty hour), and status immuniza(on. Sta(s(cal analysis was performed using the chi-square test to determine the significance of the rela(onship between employment status and exposure (me. **Result:** From 93 responden, distribution gender (60.6% female, 39.4% male), medical profession (35.5% nurse, 33.3% resident, 3.2% doctor, 23.7% medical student and 4.3% others), exposure time (53.2% duty hour, 46.8% working hour), source of exposure (61.3% needle, 34.4% blood, 5% others body fluid), 86.2% used PPE and 68,1% already had hepatitis B immunization. The analysis showed significant relationship with exposure time (p = 0.046) among medical profession and time of exposure. **Conclussion :** This study is important in identifying specific risks related to the group status of medical personnel and the time of exposure to needles, blood and body fluids to identify vulnerable workers. In addition, with this basic study, more effective safety policies and protocols can be developed by adapting to the needs of each work group. Research recommendation is needed to explore the impact of specific education and training programs in reducing the risk of exposure in HCW.

